# AI Techniques of Dermoscopy Image Analysis for the Early Detection of Skin Lesions Based on Combined CNN Features

**DOI:** 10.3390/diagnostics13071314

**Published:** 2023-04-01

**Authors:** Fekry Olayah, Ebrahim Mohammed Senan, Ibrahim Abdulrab Ahmed, Bakri Awaji

**Affiliations:** 1 Department of Information System, Faculty Computer Science and Information System, Najran University, Najran 66462, Saudi Arabia; 2 Department of Artificial Intelligence, Faculty of Computer Science and Information Technology, Alrazi University, Sana’a, Yemen; 3 Computer Department, Applied College, Najran University, Najran 66462, Saudi Arabia; iaalqubati@nu.edu.sa; 4 Department of Computer Science, Faculty of Computer Science and Information System, Najran University, Najran 66462, Saudi Arabia

**Keywords:** deep learning, ANN, fusion features, RF, skin lesion, PCA

## Abstract

Melanoma is one of the deadliest types of skin cancer that leads to death if not diagnosed early. Many skin lesions are similar in the early stages, which causes an inaccurate diagnosis. Accurate diagnosis of the types of skin lesions helps dermatologists save patients’ lives. In this paper, we propose hybrid systems based on the advantages of fused CNN models. CNN models receive dermoscopy images of the ISIC 2019 dataset after segmenting the area of lesions and isolating them from healthy skin through the Geometric Active Contour (GAC) algorithm. Artificial neural network (ANN) and Random Forest (Rf) receive fused CNN features and classify them with high accuracy. The first methodology involved analyzing the area of skin lesions and diagnosing their type early using the hybrid models CNN-ANN and CNN-RF. CNN models (AlexNet, GoogLeNet and VGG16) receive lesions area only and produce high depth feature maps. Thus, the deep feature maps were reduced by the PCA and then classified by ANN and RF networks. The second methodology involved analyzing the area of skin lesions and diagnosing their type early using the hybrid CNN-ANN and CNN-RF models based on the features of the fused CNN models. It is worth noting that the features of the CNN models were serially integrated after reducing their high dimensions by Principal Component Analysis (PCA). Hybrid models based on fused CNN features achieved promising results for diagnosing dermatoscopic images of the ISIC 2019 data set and distinguishing skin cancer from other skin lesions. The AlexNet-GoogLeNet-VGG16-ANN hybrid model achieved an AUC of 94.41%, sensitivity of 88.90%, accuracy of 96.10%, precision of 88.69%, and specificity of 99.44%.

## 1. Introduction

The largest organ in the human body is the skin. It performs many functions, such as protecting the body from external shocks, regulating temperature, protecting it from attacks by viruses and bacteria, and giving it immunity to resist diseases [1]. Its thickness varies from one area to another, ranging from 0.5 mm in the eyelids area to 4 mm in the palms of the hands [2]. It also maintains the internal organs and is considered the first line of defense, protecting the body from harmful sunlight and ultraviolet (UV) rays. It also produces vitamin D through sunlight [3]. Weather from cold to hot and skin types from oily to dry affect skin pigmentation. The sharp decrease in the levels of skin pigmentation leads to skin diseases such as skin cancer, and the cure rate is high if in the first stage. The DNA of skin cells is damaged by exposure to sunlight or ultraviolet radiation [4]. Melanoma is one of the most serious and deadly skin diseases that leads to death without an early diagnosis. Melanoma represents 1% of all types of skin cancer but causes more than 70% of deaths. According to the American Skin Cancer Society, 97,610 people have been diagnosed with melanoma, and 7990 people are expected to die [5]. It is difficult to detect skin cancer in the first stage because the cancer cells resemble the cells of the organism from which they were created. Abnormal cells multiply rapidly and abnormally through repeated cell division [6]. The cells continue to divide and multiply from one place to another, penetrating the skin layers and becoming a malignant tumor. If detected in its late stages, it causes disturbances in the functions of the skin due to its penetration into the lower layers where the blood vessels are located, which requires painful surgeries with a low survival rate [7]. The skin consists of three layers: the epidermis, the dermis, and the hypodermis. The epidermis is the body’s outer layer and protects the body from all infections and does not contain blood vessels. The epidermis consists of keratinocytes, Merkel cells with Langerhans, and melanocytes [8]. These cells are a powerful barrier to infection. The dermis is the layer under the epidermis that contains nerve endings sensitive to heat and touch. The hypodermis lies under the dermis and contains nerves and blood vessels and is responsible for storing fat. Dermoscopy is a non-invasive technique for detecting pigment, patterns, pigment mesh, shape, color and combinations. This allows dermatologists to diagnose skin lesions better than with the naked eye [9]. Dermatologists identify skin lesions by analyzing dermatoscopy images and comparing the lesion characteristics to detect the type of lesion for the patient to receive the appropriate treatment. The similarity of features and clinical signs of early lesions and the different opinions of dermatologists represent a challenge for the early diagnosis of melanoma [10]. There is also a significant gap between the number of patients and experienced dermatologists. The development of computer processors has played a vital role in all fields, including healthcare, for biomedical image diagnostics. Computer-assisted medical diagnosis is an important and modern study to help experts, specialists, and doctors to perform medical image analysis for early and effective diagnosis and to save time and effort in detecting diseases. Deep learning techniques are applied to many medical, dermatoscopic, radiological, and MRI images to extract features accurately. Medical image quality is of fundamental importance for effective diagnosis. Medical image processing provides filters to increase image quality and eliminate noise and artifacts. This study focused on the weaknesses, such as the similarity of characteristics between skin lesions, to reach promising results for diagnosing dermoscopic images. Effective techniques have been developed based on hybrid features of several methods to diagnose dermoscopic images effectively.

The major contributions to this study are as follows:

- Improving dermatoscopy images using two successive techniques: CLAHE and average filter
- Segmentation of dermatoscopy images of the ISIC 2019 dataset using the GAC algorithm and then feeding them to CNN models
- Analysis of dermatoscopy images for early diagnosis of skin cancer and their distinction from skin lesions by hybrid models CNN-ANN and CNN-RF based on the GAC algorithm
- Analysis of dermatoscopy images for the early diagnosis of skin cancer and distinguishing them from skin lesions using the ANN and RF networks based on the fused CNN features.

The rest of the paper is organized as follows: Section 2 discusses techniques and findings from previous studies. Section 3 presents methods for analyzing dermatoscopic images for the early diagnosis of skin lesions. Section 4 presents the findings of the hybrid models. Section 5 discusses the results of the systems and compares their performance. Section 6 concludes the research.

## 2. Related Work

Ismail et al. [11] proposed an EfficientNet-B6 model for diagnosing the images of the ISIC 2020 data set as malignant or benign with an accuracy of 97.84%. Since malignant lesions represent 2% of the data set, data oversampling and over-sampling techniques were applied to address the imbalance in the data set. Federico et al. [12] analyzed the impact of CNN structures on the accuracy of ISIC 2018 image analysis, data augmentation, and parameter calibration. The method achieved an accuracy of 59.3% for classifying the ISIC 2019 data set. Sun et al. [13] applied a Grad-CAM method to generate a heat-map for ISIC 2018 image diagnostics along with metadata. Metadata contains patient data, lesion location, age, and sex. The method achieved an accuracy of 88.7% and an accuracy with metadata of 89.5%. Mark et al. [14] used CNN uncertainty estimation methods based on Monte Carlo samples to solve the ISIC 2019 dermoscopic image classification problem. The method proved its effective accuracy in detecting impossible to distribute and difficult images. The approach reached an accuracy of 76% for classifying the ISIC 2018 data set. Gong et al. [15] developed a decision fusion method based on pre-trained CNNs. Multiple networks are integrated through the block, and the multiple blocks make the decision. StyleGANs are used to improve image quality and improve CNN classification. The VGG16BN achieved an accuracy of 95.6%, a sensitivity of 69.2%, a specificity of 96.9%, and an AUC of 89.6%. Tryan et al. [16] developed an EfficientNet dynamic training network to dramatically increase the performance of ISIC 2018 dataset image diagnostics. Bayesian optimization was used to train the model further. The model achieved an accuracy of 95%, a sensitivity of 65%, an F1-score of 64%, and an AUC of 91%. Tryan et al. [17] applied an ensemble approach based on two CNN models to detect melanoma. The texture features were extracted, dimensionally reduced, and combined with CNN models. The method reached an accuracy of 96.7%, a sensitivity of 95.1%, and a specificity of 96.3% for diagnosing images of the ISIC 2019 dataset. Imran et al. [18] presented a DCNN model with various layers and different filter sizes to improve the model’s performance for ISIC 2019 image diagnostics. The DCNN achieved an accuracy of 94%, a sensitivity of 93%, and a specificity of 91%. Krishna et al. [19] presented MSVM algorithms for skin lesions classification. The hair was removed by the Dull razor method and the average filter for more enhancement. Use the k-means method to segment the disease area and extract the features using GLCM and ABCD methods. The MSVM achieved an accuracy of 96.25%. Tri et al. [20] proposed improving the InceptionV3 and Resnet50 models to train the images of the ISIC 2019 dataset and test its performance based on 10% of the dataset. The models solved the problem of overfitting and outperformed 153 physicians in classification accuracy. The InceptionV3 attained a sensitivity of 70%, a specificity of 91%, and an AUC of 88.4%. Long et al. [21] proposed an approach to lesion segmentation using the EW-FCM method and ShuffleNet for its classification. The EW-FCM-ShuffleNet method achieved good performance in ISIC 2019 dataset image classification. The EW-FCM + EfficientNet-B0 model attained an accuracy of 84.66%, a sensitivity of 84.669%, and a specificity of 97.81%. Junsheng et al. [22] developed a segmentation network based on the co-occurrence area to exclude the healthy part and send it to the inference unit for lesions segmentation. The ResNet-50 achieved an accuracy of 68.4%, a sensitivity of 58.69%, and a specificity of 70.1%. Hadi et al. [23] applied machine learning based on CNN to identify skin lesions. The lesion area was extracted and fed to the CNN models, which achieved better results than feeding the CNN models with the full image, with an improvement rate of 2.18%. Mohamed et al. [24] applied the pre-trained GoogleNet model based on modifiable parameters during training. The model achieved an accuracy of 94.92%, a sensitivity of 79.8%, and a specificity of 97% for classifying eight classes of the ISIC 2019 dataset. Juan et al. [25] propose three CNN models to classify dermoscopy images for the HAM10000 and ISIC 2018 datasets, and their results are compared. Inception-V3 achieved 96% and 93% accuracy for the HAM10000 and ISIC 2018 datasets, respectively.

The researchers focused on effectively classifying dermatoscopic images of the ISIC 2019 dataset using CNN models and machine learning but did not reach satisfactory accuracy. Additionally, previous studies lacked the application of hybrid methods based on fused characteristics, which is the backbone for addressing the similarity of clinical signs and reaching promising results. This study was characterized by the diversity of hybrid methods and tools between machine and deep learning based on features fused between several CNN models.

## 3. Materials and Methods

### 3.1. Description of ISIC 2019 Dataset

The proposed models were trained and tested on the publicly available ISIC 2019 dataset for researchers interested in diagnosis and prediction. The ISIC 2019 dataset contains 25,331 images from the HAM10000 and BCN_20000 datasets. The dataset provides high-resolution images with metadata of lesion location, sex, and age of the infected. The dataset consists of 10015 RGB images with a resolution of 600 × 450 pixels, while the BCN_20000 dataset contains RGB images with a resolution of 1024 × 1024 pixels [26]. The ISIC 2019 dataset is distributed over eight classes (types of skin lesions) as follows: 628 images of Squamous cell carcinoma (Scc), 867 images of Actinic keratoses (Akiec), 3323 images of Basal cell carcinoma (Bcc), 2624 images of Benign keratosis lesions (Bkl), 239 images of Dermatofibroma (Df), 4522 images of Melanoma (MEL), 12,875 images of Nevi (VN) and 253 images of Vascular (Vasc). The dataset was used on different hybrid technologies based on hybrid characteristics. This work aims to detect the type of skin lesion early and distinguish melanoma from other malignant skin lesions.

### 3.2. Enhancement of ISIC 2019 Dermoscopic Images

The first step for all proposed systems is to improve dermatoscopic images. The Dermatoscopy images include noise and artifacts due to the variety of acquisition devices, which negatively affect the subsequent stages of image processing and lead to unreliable results [27]. So, the main purpose of pre-processing is to remove noise and artifacts such as air bubbles, hair, skin lines, low contrast between lesion borders, and light reflections when the gel is applied to the skin at the time of image capture. RGB channels were averaged, and color constancy was adjusted [28].

The presence of some artifacts leads to the occlusion of an essential part of the skin lesion or the extraction of false features, which makes the extracted features incorrect. A 6 by 6 averaging filter was applied to refine the images of the ISIC 2019 dataset, and the pixel value adjacent to the unwanted pixel was calculated using a 2D convolution operator. On each iteration, the operator targets a convolution of 36 pixels divided into one target pixel and 35 adjacent pixels. The average of adjacent pixels is calculated, and its value is substituted for the target pixel as in Equation (1) [29].

(1) $$F\left(x\right)=\frac{1}{M}\overset{M-1}{\underset{i=0}{\sum }}z\left(x-i\right)\;$$

where *F*(*x*) refers to the output, M refers to the number of pixels in operators, *z*(*x*) refers to the input and *z*(*x* − *i*) refers to the prior input.

**Figure 1:**
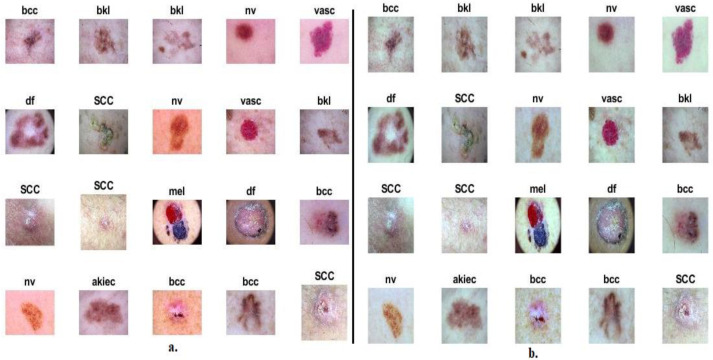
Sample dermatoscopy images of the ISIC 2019 dataset (**a**) before improvement (**b**) after improvement.

Due to the low contrast between the edges of the lesions and their periphery, the low contrast was improved by the contrast limited adaptive histogram equalization (CLAHE) technique. The basic idea of this technique is to distribute the bright pixels to dark regions based on neighboring pixels. Each pixel is compared with its neighbors in each iteration and based on the comparison, the contrast is improved as follows: if the value of the target pixel is greater than its neighbors, the contrast will be reduced. In contrast, the image contrast increases when the neighboring pixels are greater than the target pixel [30]. Thus, the mechanism continues for each pixel in the image until the appearance of the edges of the skin lesions is improved. Figure 1b shows samples from the ISIC 2019 dataset after undergoing optimization techniques.

### 3.3. Geometric Active Contour Algorithm

The Geometric Active Contour (GAC) algorithm is a contour model used to segment biomedical images such as skin lesions, extract a region of interest (ROI), and separate it from the healthy regions [31]. The algorithm creates a set of points and moves them on the perpendicular curve of the lesion edges to obtain a smooth curve. So that the movement of the points on the curve is proportional to the curves in the ROI in the image. The contour is described based on the geometric flow of the curves to detect the lesion area [32]. The engineering flows are conducted according to the external and internal measurements of the ROI. By the geometric flow of the contour, the geometric lines of the initial curve C0 are determined as in Equation (2).

(2) $$Ct=g\left(C\right)\left(k-v\right)\left|N^{\to }\right|\;$$

where *g* refers to the edge scalar function, *k* refers to a vector of curvature, $N^{\to }$ refers to from the vector to the curve and *v* refers to a constant value.

The curve continues to move until g reaches zero, which means the curve has reached the edge of the skin lesion. When the curve comes to the edge of the lesion, the parameters are replaced by the Euclidean arc length as in Equation (3).

(3) ds=|Cp|dp 

Euclidean arc length explains irregular curves based on curves and energy forces. Minimal geometric curve flows are derived through internal and external forces. Equation (4), provided by Euler-Lagrange, shows the curved differential for the ROI.

(4) $$\frac{dC}{dt}=\left(g\left(C\right)k-\langle \nabla g,N^{\to }\rangle \right)N^{\to }\;$$

The lesion area is determined based on the geometric plane developing curve functions. The minimum internal force is applied through the force of the balloon, which shows progress in the inner circumference of the lesion. Thus, the Euler-Lagrange expression defines the contour as the innermost descending as in Equation (5) [33].

(5) dCdt=gCk−∇gN→−σgCN→ 

**Figure 2:**
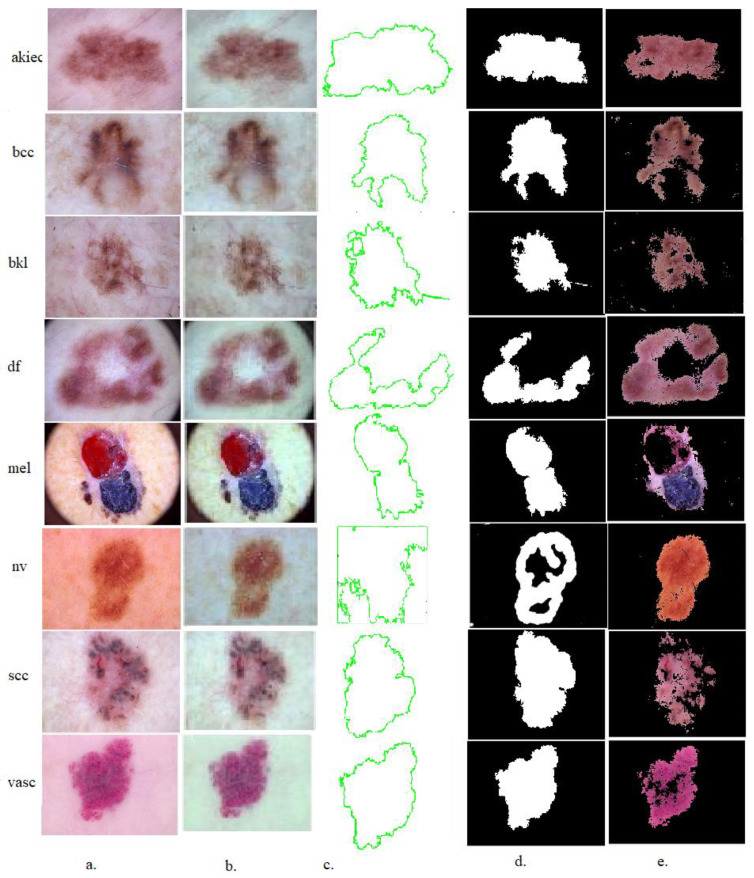
A sample from each class of the ISIC 2019 dataset for extracting the lesion area and its isolation from the healthy skin using the GAC method (**a**) Original images (**b**) Enhanced images (**c**) Selection of the lesion area (**d**) Segmentation of the lesion area (**e**) Lesion area (ROI).

The contour models describe the curve of the geometric flows to show the geometric features of the edges of the lesion area as shown in Figure 2. The edges of the skin lesions are defined based on the color gradation of the edges by the active geometric lines. Edge-based engineering models have effective computational capabilities to segment lesions areas. There are gaps in some areas due to the graduated curves. Therefore, the engineering models are sensitive to the graduated curves to determine the contour by increasing the weights of the curves and the horizontal width. Geometric models depend on the difference in density inside and outside the contour lines or lesions’ internal and external contrast.

In this study, the skin lesion regions were segmented and stored in a new folder to be sent to the CNN models to extract features from the lesion regions only (ROI) instead of feeding the CNN models with full dermoscopic images of the diseased and healthy parts.

### 3.4. Extract Deep Feature Maps

CNNs consist of many convolutional and pooling layers ending in a series of fully connected layers. The convolutional layers receive an image of size *m* × *n* × *z*, where m and n are the width and height of the image and z is the number of color channels [34]. The number of convolutional layers differs from one network to another, and each convolutional layer usually consists of many filters of size *f* × *f* × *z*. The number of channels of the input image must match the number of channels of the convolutional filter. The key to convolutional neural networks is the convolution process of superimposing the filter *f*(*t*) on the input image *x*(*t*) and moving it over the image until all areas of the image are processed (the filter must process all pixels of the image) as in Equation (6) [35].

Each convolutional layer produces feature maps equal to the number of filters in the layer and the new image becomes an entry to the next layer after adding some biases and passing it from auxiliary layers such as ReLU.

(6) y(t)=(x∗f)(t)=∫x(a)f(t−a) da 

where *f*(*t*) denotes the filter, *x*(*t*) denotes the image input and *y*(*t*) denotes output.

After convolutional layers, the feature maps are very dimensional, so the data size must be reduced. CNN networks provide pooling layers of two types, either max or average, which work to reduce the size of the input images through the implementation of the pooling process [36]. The pooling layers interact similarly to the convolutional layer, which produces operations in small regions of the input matrix area. The Max-Pooling works by selecting a group from the size of the matrix and searching for the max value and replacing it with the greatest value as in Equation (7). The average-Pooling work mechanism is the same as the Max-Pooling work mechanism, except instead of replacing the selected group with max here, it is replaced by the arithmetic average for the selected group as in Equation (8).

Thus, the computational cost is reduced, as pooling results in an output matrix with dimensions much smaller than the output matrix of the convolutional layer. At the same time, it helps to obtain and locate dominant features. CNN networks have achieved great success in identifying features from the input images and their high ability to detect complex features effectively because filters act as detectors for hidden and small features such as edges, shapes, colors, structures, and so on [37].

(7) $$z\left(i;\;j\right)=max_{m,n=1\ldots .k}\;f\left[\left(i-1\right)p+m;\;\left(\;j-1\right)p+n\right]\;$$

(8) $$z\left(i;\;j\right)=\frac{1}{k^{2}}\underset{m,n=1\ldots .k}{\sum }f\left[\left(i-1\right)p+m;\;\left(\;j-1\right)p+n\right]\;$$

where *f* indicates the wrapped filter in area image, *m*, *n* indicates the matrix location, *k* indicates number of pixels and p indicates p-step.

Finally, the output should be a classification of the inputs (probabilities for each class), high-level features are converted to vectors by fully connected layers [38]. Followed by the SoftMax activation function, which consists of neurons with a number of input classes. SoftMax works on labelling the features of each image to its appropriate class. CNN is computationally efficient in that the features in one area of the image are often the same as those in another part of the image, which provides the use of the same weights to calculate activations on other regions of the image. Thus, the number of parameters, weights, and links to be trained is reduced [39].

The last convolutional or pooling layers of the AlexNet, GoogLeNet and VGG16 models produce higher-level feature maps as follows: (13, 13, 256), (7, 7, 512) and (7, 7, 512), respectively. Finally, the high-level features are converted to features vectors using a global average pooling layer, which puts the high-level features into flat vectors of size 4096 for each model AlexNet, GoogLeNet and VGG16. Thus, the size of the ISIC 2019 dataset is represented by a feature matrix with the size 25,331 × 4096 for AlexNet, GoogLeNet and VGG16 separately.

### 3.5. Inductive and Deductive Phase

The last stage of medical image processing is classification, which depends on the previous stages’ efficiency. After the stages of optimization and extraction of the ROI (lesion area), the features of the skin lesions are extracted by CNN models and saved in vectors. The data set’s features are represented in the feature matrix, which is input to the ANN and RF networks. Classification networks include an inductive phase to build a classification model called data training and the deductive stage of testing new data to measure the system’s performance.

#### 3.5.1. ANN Network

**Figure 3:**
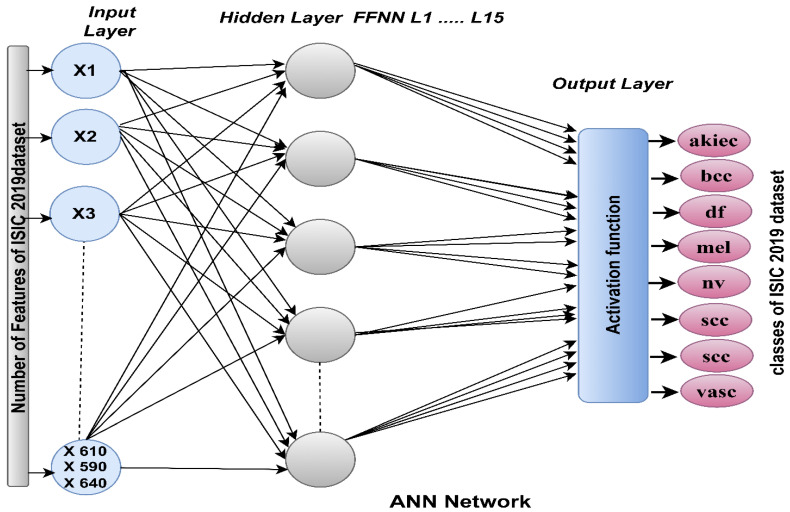
ANN structure for analyzing dermatoscopic images to diagnose the skin lesions of the ISIC 2019 dataset.

The ANN is a type of high-efficiency soft computing. ANN consists of three basic layers connected by many interconnected neurons with exact weights. The network can effectively extract information from complex data, analyze it, produce clear and interpretable patterns, and adapt to changing environments. The ANN passes information between layers and neurons and reduces the computational error probability of overlapping affinities between classes. ANN consists of processing units that send signals to each other through weighted links. Each neuron has an activation unit using weight wjk by signal j on the k unit. It also has a spreading base that receives external inputs and determines effective ones. The ANN input layer receives feature vectors extracted by CNN models and hybrid features between CNN models [40]. The input layer consists of units with the same number of features extracted from the previous stage. The inputs of the input layer are passed to the hidden layers in which the calculations are performed to perform the required tasks. The accuracy of the new data classification depends on the system’s performance when building the training and validation template. The performance of an ANN depends on its internal structure, the number of hidden layers, and its neurons. In this study, the number of hidden cells was set to 15 hidden layers, as shown in Figure 3. The network measures its performance through squared errors between the actual xi and expected zi values. The network is repeated, and in each iteration, the weights are set iteratively until the network reaches an optimal set of weights through minimum square error (MSE), as in Equation (9). The output layers contain eight neurons equal to the dataset’s number of classes. The activation function in the output layer maps all feature vectors to their appropriate category.

(9) $$\;\mathrm{MSC}=\frac{1}{m}\overset{m}{\underset{i=1}{\sum }}\;{\left(\;x_{i}-z_{i}\right)}^{2}\;$$

where m means the number of data, $x_{i}$ means actual output, and $z_{i}$ means expected output.

#### 3.5.2. Random Forest Network

The random forest algorithm has a superior ability to make effective predictions on a biomedical dataset. As its name suggests, it is built based on assembling the predictions of many trees. RF aggregates the results of each decision tree and makes a decision based on majority voting, which is called the ensemble learning method. RF selects data points randomly, and based on these points, the algorithm creates decision trees with a specified number of points. RF uses its hyperparameters to increase the predictive efficiency of the classifier. More decision trees increase the performance and stability of predictions, but they increase the processing time after achieving the training step and creating a training model that can be applied to a new data set to test the system and measure its generalization on new data. RF works with the Bagging method based on creating a sub-set of data and the final decision based on a majority vote for all decision-making trees. The mechanism begins with random data known as bootstrap data and called Bootstrapping. The models are trained separately, and each decision tree produces a result. In the end, the results are collected, called aggregation, and decisions are made according to the majority vote.

This study used CNN networks (AlexNet, GoogLeNet and VGG16) to extract deep feature maps from ISIC 2019 dermoscopy images and classify them using ANN and Random Forest networks. It is worth noting that the performance of the hybrid systems was evaluated using ISIC 2019 images before and after applying the GAC algorithm to extract the ROI.

**Figure 4:**
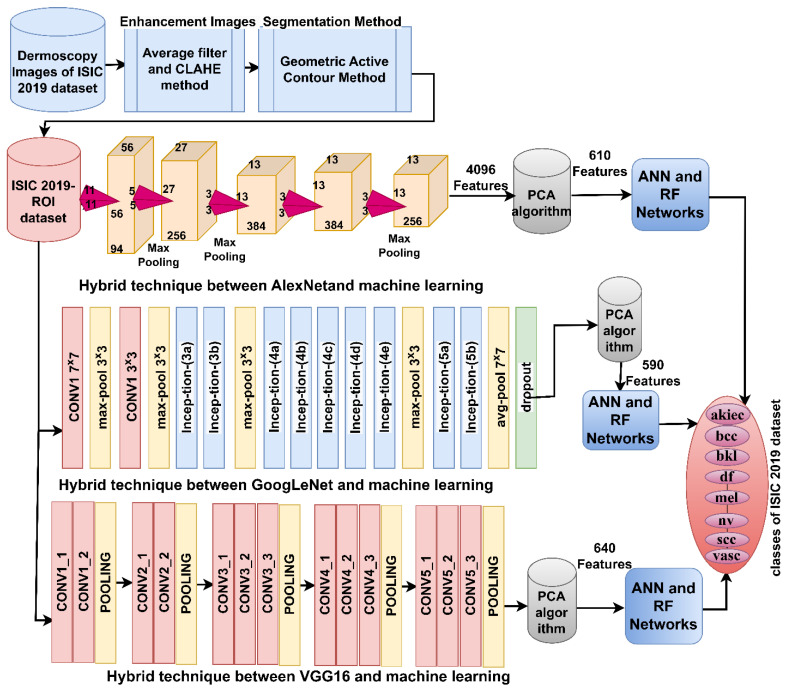
A hybrid model of CNN and machine learning for analyzing dermatoscopic images to diagnose the skin lesions of the ISIC 2019 dataset.

The ISIC 2019-ROI image classification strategy using a hybrid model of CNN-machine learning based on the GAC segmentation algorithm goes through the following implementation steps, as shown in Figure 4. First, the ISIC 2019 dataset images was improved to remove artifacts. Second, the lesion area was segmented and separated from healthy skin by the GAC algorithm and stored in a new folder called ISIC 2019-ROI. Third, the images of the new ISIC 2019-ROI dataset were fed into AlexNet, GoogLeNet and VGG16 models separately. Feature maps were extracted from each model by convolutional layers and pooling and saved as a feature matrix of sizes 25,331 × 4096, 25,331 × 4096, and 25,331 × 4096 for AlexNet, GoogLeNet and VGG16 models. Fourth, the high-dimensional feature matrix was fed into PCA to remove non-significant and redundant features and retain the most representative features. The PCA method produced a highly representative feature matrix of sizes 25,331 × 610, 25,331 × 590, and 25,331 × 640 for AlexNet, GoogLeNet, and VGG16 models, respectively. Fifth, the representative features matrix was fed to the ANN and RF networks to train and test their performance.

**Figure 5:**
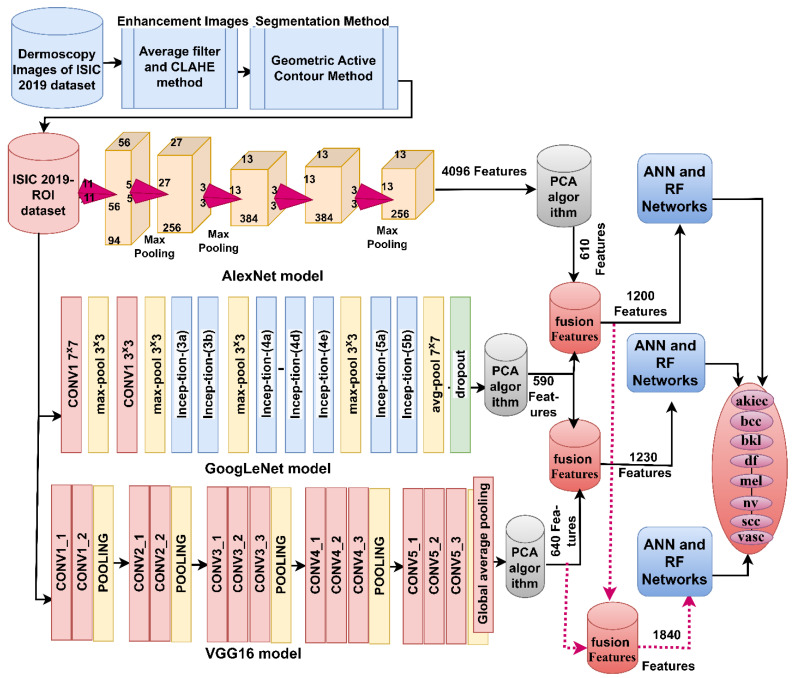
A hybrid model of ANN and RF with fused features of CNN for analyzing dermatoscopic images to diagnose the skin lesions of the ISIC 2019 dataset.

The ISIC 2019-ROI image classification strategy by machine learning classifiers with combined features of CNN models goes through the following implementation steps as shown in Figure 5: The first four implementation steps are the same as the previous strategy. Fifth, the deep feature maps combine the CNN models Serially: AlexNet-GoogLeNet, GoogLeNet-VGG16, AlexNet-VGG16 and AlexNet-GoogLeNet-VGG16. Thus, the fused feature matrix has sizes of 25,331 × 1200, 25,331 × 1230, 25,331 × 1250 and 25,331 × 1840 for each of AlexNet-GoogLeNet, GoogLeNet-VGG16, AlexNet-VGG16 and AlexNet-GoogLeNet-VGG16 models, respectively. Sixth, the highly representative feature matrix is fed to the ANN and RF networks to train and test their performance.

## 4. Experimental Results of the Proposed Systems

### 4.1. Split of ISIC 2019 Data Set

The performance of the proposed systems in this study was measured on the dermatoscopic images available online to researchers and experts of the ISIC 2019 dataset. The ISIC 2019 dataset contains 25,331 dermatoscopic images distributed unevenly among eight classes (types of skin diseases), melanocytic and skin non-Melanocytic types, as shown in Table 1. In all proposed strategies, the data set was randomly divided into 80% during training and validation (80:20) and 20% for the testing phase. As shown in the table, the data set is unbalanced, highlighting a problem that needs to be addressed.

### 4.2. Systems Performance Measures

The confusion matrix is one of the most important standard tools that evaluates the performance of systems for classifying a data set. The confusion matrix is a quadrilateral matrix with an equal number of rows and columns based on the data set classes. The confusion matrix contains the number of correctly and incorrectly classified test group samples. The main diagonal represents correctly classified samples called true positive (TP), and the rest of the cells represent incorrectly classified samples called true negative (TN) and false negative (FN). The performance of the systems is measured through Equations (10)–(14). The equations derive their variables from the confusion matrix [41].

(10) $$\mathrm{AUC}\;=\frac{\mathrm{TP}\;\mathrm{Rate}}{\mathrm{FP}\;\mathrm{Rate}}\;$$

(11) $$\mathrm{Sensitivity}=\frac{\mathrm{TP}}{\mathrm{TP}+\mathrm{FN}}\;*\;100\%\;$$

(12) $$\mathrm{Accuracy}=\frac{\mathrm{TN}+\mathrm{TP}}{\mathrm{TN}+\mathrm{TP}+\mathrm{FN}+\mathrm{FP}}\;*\;100\%$$

(13) $$\mathrm{Precision}=\frac{\mathrm{TP}}{\mathrm{TP}+\mathrm{FP}}\;*\;100\%$$

(14) $$\mathrm{Specificity}=\frac{\mathrm{TN}}{\mathrm{TN}+\mathrm{FP}}\;*\;100\;$$

### 4.3. Balancing Classes of ISIC 2019 Dataset

**Figure 6:**
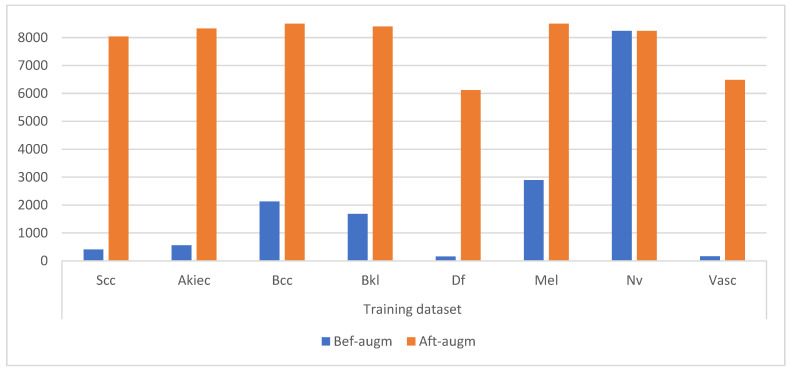
Display the number of dermoscopy images of the ISIC 2019 data set before and after data augmentation.

CNN models face many challenges, including the problem of overfitting, because they need a very large data set, which is not available in the biomedical data set. Additionally, the unbalanced data set, which contains unbalanced classes, is a challenge for the results of artificial intelligence models because the accuracy tends to class which has the majority images. Thus, CNN models provide a data augmentation tool to address these challenges. The tool increases the training data set’s images from the original data set through many operations, such as rotating the images at various angles, resizing, vertical and horizontal transformation, vertical and horizontal flipping, displacement, shearing, and others [42]. To obtain balanced classes, the images were increased in seven categories, while the nevi class was not increased because it contained sufficient images. Additionally, the images of each class have been increased by a different amount from the other classes to achieve a balance. Table 2 and Figure 6 shows the number of images of the ISIC 2019 training data set before and after applying the data augmentation tool. Where it is noted that each image in the Scc class increased by 20 times, each image in the Akiec class increased by 15 times, each image in the Bcc class increased by 4 times, each image in the Bkl class increased by 5 times, each image in the Df class increased by 40 times, each image in the Mel class increased by 3 times, and each image in the Vasc class increased by 40 times.

### 4.4. Results of Pre-Trained Deep Learning

This section summarizes the performance results of the pre-trained AlexNet, GoogLeNet and VGG16 models. These models were trained on the ImageNet dataset, which has more than 1,200,000 images to classify more than 1000 classes. Unfortunately, the ImageNet dataset does not contain most biomedical image datasets, such as dermatoscopic images of skin lesions. These models transfer the experience gained when training the ImageNet dataset to perform new dermatoscopic image classification tasks. The input layers receive the skin lesions images of the ISIC 2019 dataset and send them to the convolutional, pooling and auxiliary layers for processing and extracting the deep and hidden features. Fully-connected layers convert higher-level features into vectors and classify each feature vector into an appropriate class.

**Figure 7:**
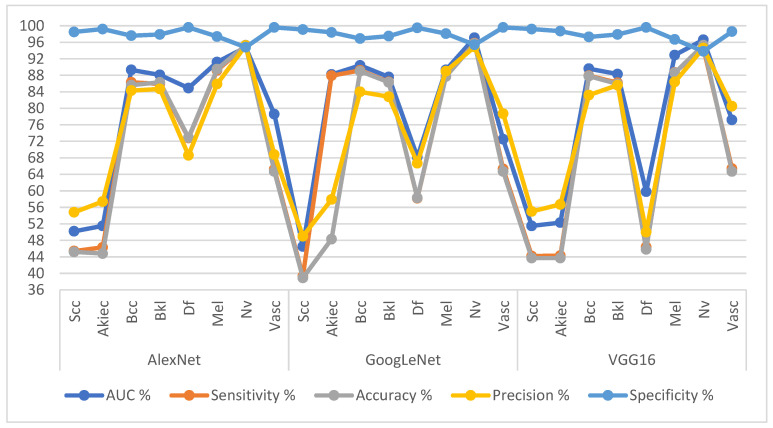
Display performance results of pre-trained CNN models for analysis of the ISIC 2019 dataset image for early diagnosis and distinction of skin cancer and other skin lesions.

Table 3 and Figure 7 summarize the results of AlexNet, GoogLeNet and VGG16 pre-trained models for analyzing dermatoscopic images to diagnose the ISIC 2019 dataset. The AlexNet achieved average results: AUC of 78.58%, sensitivity of 73.29%, accuracy of 88.0%, precision of 74.96%, and specificity of 98.08%. The GoogLeNet achieved average results: an AUC of 79.98%, sensitivity of 76.23%, accuracy of 88.70%, precision of 75.38%, and specificity of 98.06%. The VGG16 achieved average results: an AUC of 76.03%, sensitivity of 69.71%, accuracy of 88.30%, precision of 69.44%, and specificity of 97.73%.

### 4.5. Results of Pre-Trained Deep Learning Based on GAC Algorithm

In this section, we summarize the performance results of pre-trained AlexNet, GoogLeNet and VGG16 models based on the segmentation of dermatoscopy images using the GAC algorithm. The dermatoscopy images of the ISIC 2019 dataset were first segmented, and only the lesions area was extracted and saved in new folders to be fed into AlexNet, GoogLeNet and VGG16 models. The input layers receive the segmented skin lesions images of the ISIC 2019 data set and send them to the convolutional, pooling and auxiliary layers for processing and extracting the deep and hidden features. Fully-connected layers convert higher-level features into vectors and classify each feature vector into an appropriate class.

**Figure 8:**
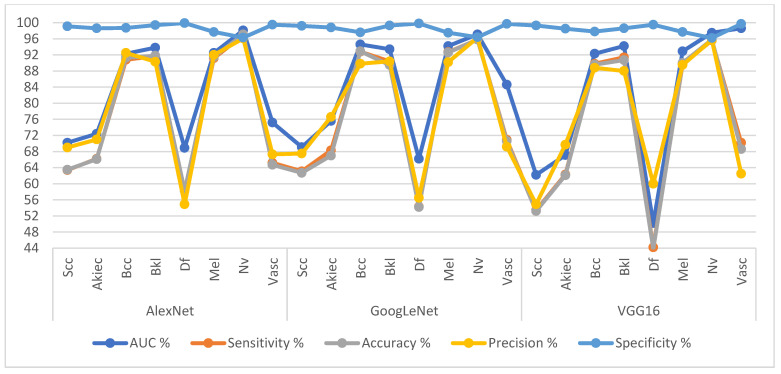
Display Performance results of CNN models based on ROI using the GAC method for early diagnosis and distinction of skin cancer and other skin lesions.

Table 4 and Figure 8 summarize the results of the AlexNet, GoogLeNet and VGG16 models based on the GAC algorithm for dermatoscopic image analysis for diagnosis of the ISIC 2019 dataset. The AlexNet achieved average results: an AUC of 82.93%, sensitivity of 77.96%, accuracy of 92.20%, precision of 79.14%, and specificity of 98.65%. GoogLeNet has achieved average results: an AUC of 84.34%, sensitivity of 87.54%, accuracy of 91.80%, precision of 79.56%, and specificity of 98.54%. VGG16 has achieved average results: an AUC of 81.88%, sensitivity of 74.58%, accuracy of 90.50%, precision of 76.14%, and specificity of 98.41%.

### 4.6. Results of Hybrid Models of CNN, ANN and RF

This section discusses the summary results of hybrid models between CNN models (AlexNet, GoogLeNet and VGG16) with both ANN and RF networks separately for image analysis of the ISIC 2019 dataset for skin lesions. The mechanism of the hybrid models is segmentation of the lesion area after image optimization and feature map extraction through CNN models. To keep the important features and delete the redundant ones using the PCA method, the feature vectors generated by PCA are sent to the ANN and RF networks for training and performance testing.

The CNN-ANN and RF-CNN hybrid models for image analysis of skin lesions of the ISIC 2019 dataset have high capabilities in distinguishing skin cancer from other skin diseases.

**Figure 9:**
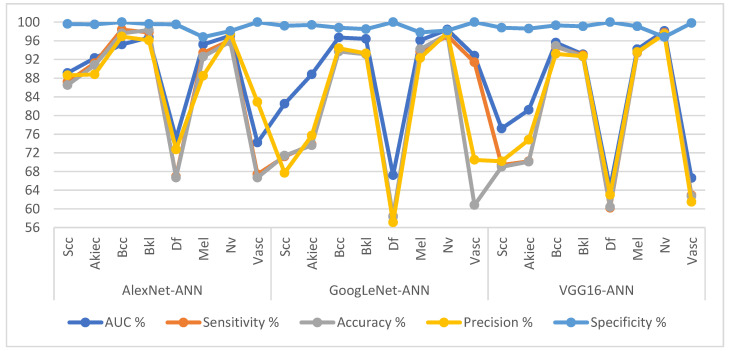
Display Performance results of CNN-ANN hybrid models based on ROI using the GAC method for early diagnosis and distinction of skin cancer and other skin lesions.

Table 5 and Figure 9 present the measurement performance of the CNN-ANN hybrid models for the ISIC 2019 dataset image analysis for early diagnosis of skin lesions as follows. The AlexNet-ANN model achieved average results: an AUC of 89.36%, sensitivity of 89.36%, accuracy of 94.80%, precision of 89%, and specificity of 99.14%. The GoogLeNet-ANN model achieved average results: an AUC of 89.86%, sensitivity of 84.09%, accuracy of 93.7%, precision of 81.09%, and specificity of 98.99%. The VGG16-ANN model yielded average results: an AUC of 83.81%, sensitivity of 80.18%, accuracy of 93.80%, precision of 100%, and specificity of 98.94%.

**Figure 10:**
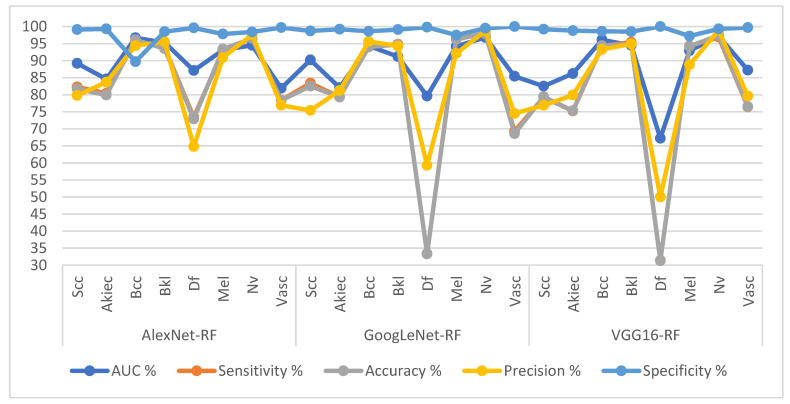
Display Performance results of CNN-RF hybrid models based on ROI using the GAC method for early diagnosis and discrimination of skin cancer and other skin lesions.

Table 6 and Figure 10 present the measurement performance of the CNN-RF hybrid models for the ISIC 2019 dataset image analysis for the early diagnosis of skin lesions as follows. The AlexNet-RF model achieved average results: an AUC of 90.30%, sensitivity of 86.69%, accuracy of 94.30%, precision of 85.45%, and specificity of 97.76%. The GoogLeNet-RF model achieved average results: an AUC of 89.23%, sensitivity of 81.09%, accuracy of 94.90%, precision of 83.89%, and specificity of 99.05%. The VGG16-RF model yielded average results: an AUC of 87.98%, sensitivity of 80.40%, accuracy of 94.20%, precision of 82.79%, and specificity of 98.91%.

The hybrid models of CNN-ANN and RF-CNN produce confusion matrices that show the performance of the hybrid models for the early detection of skin cancer for distinguishing skin cancer from other lesions.

**Figure 11:**
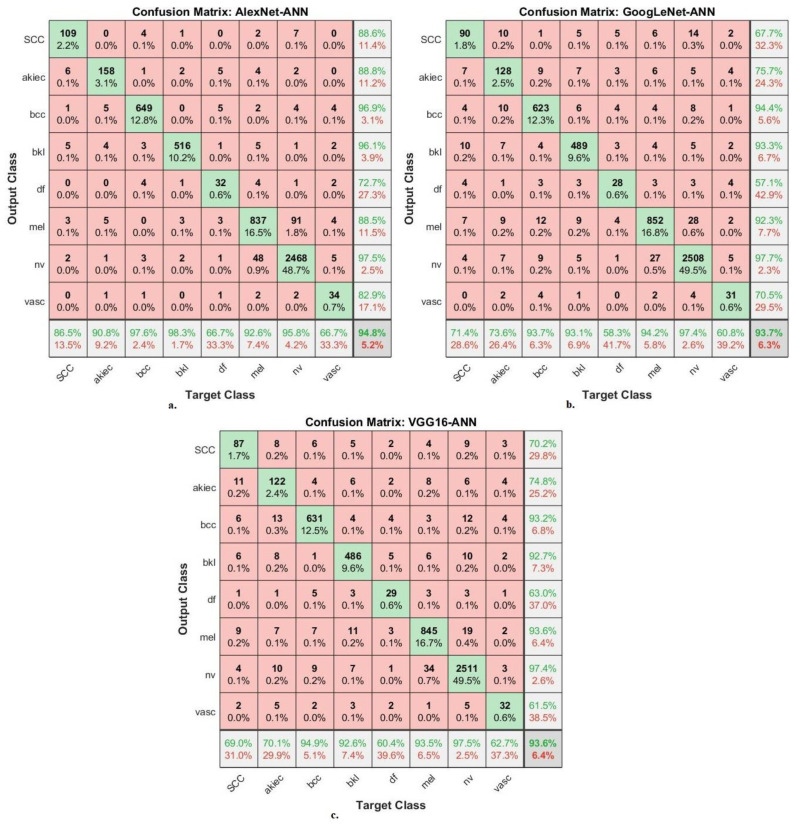
Confusion matrix for displaying performance results of hybrid CNN-ANN models based on ROI using the GAC method for early diagnosis and discrimination of skin cancer from other skin lesions (**a**) AlexNet-ANN (**b**) GoogLeNet-ANN (**c**) VGG16-ANN.

Figure 11 presents the confusion matrix of the AlexNet-ANN, GoogLeNet-ANN and VGG16-ANN models for early diagnosis of skin lesions of the ISIC 2019 data set. The figure displays the accuracy of each class, where the AlexNet-ANN model reached an accuracy for each class as follows. For Scc class of 86.5%, for Akice class of 90.8%, for Bcc class of 97.6%, for Bkl class of 98.3%, for Df class of 66.7%, for Mel class of 92.6%, for Nv class of 95.8%, and Vasc class of 66.7%. The GoogLeNet-ANN model achieved accuracy for each class as follows: for Scc class of 71.4%, for Akice class of 73.6%, for Bcc class of 93.7%, for Bkl class of 93.1%, for Df class of 58.3%, for Mel class of 94.2%, for Nv class of 97.4%, and Vasc class of 60.8%. The VGG16-ANN model resulted in accuracy for each class as follows: for Scc class of 69%, for Akice class of 70.1%, for Bcc class of 94.9%, for Bkl class of 92.6%, for Df class of 60.4%, for Mel class of 93.5%, for Nv class of 97.5%, and Vasc class of 62.7%.

**Figure 12:**
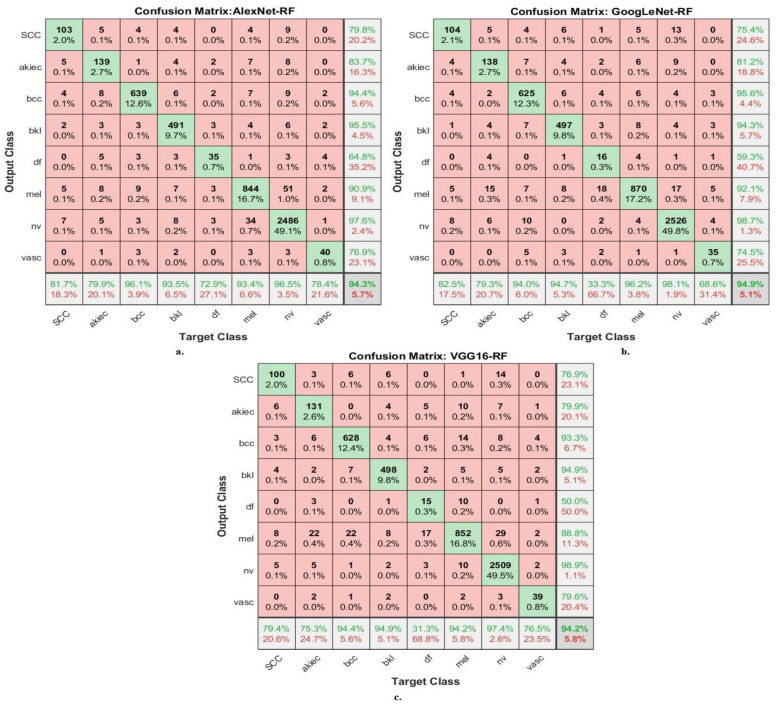
Confusion matrix for displaying performance results of hybrid CNN-RF models based on ROI using the GAC method for early diagnosis and discrimination of skin cancer from other skin lesions (**a**) AlexNet-RF (**b**) GoogLeNet-RF (**c**) VGG16-RF.

Figure 12 presents the confusion matrix of the AlexNet-RF, GoogLeNet-RF and VGG16-RF models for the early diagnosis of skin lesions of the ISIC 2019 data set. The figure displays the accuracy of each class, where the AlexNet-RF model reached an accuracy for each class as follows: for Scc class of 81.7%, for Akice class of 97.9%, for Bcc class of 96.1%, for Bkl class of 93.5%, for Df class of 72.9%, for Mel class of 93.4%, for Nv class of 96.5%, and Vasc class of 78.4%. The GoogLeNet-RF model achieved accuracy for each class as follows: for Scc class of 82.5%, for Akice class of 79.3%, for Bcc class of 94%, for Bkl class of 94.7%, for Df class of 33.3%, for Mel class of 96.2%, for Nv class of 98.1%, and Vasc class of 68.6%. The VGG16-RF model resulted in accuracy for each class as follows: for Scc class of 79.4%, for Akice class of 75.3%, for Bcc class of 94.4%, for Bkl class of 94.9%, for Df class of 31.3%, for Mel class of 94.2%, for Nv class of 97.4%, and Vasc class of 76.5%.

### 4.7. Results of Hybrid Models Based on Fused CNN Features

This section presents the findings of hybrid models based on CNN (AlexNet, GoogLeNet and VGG16) features fused to image analysis of the ISIC 2019 dataset for the early diagnosis and discrimination of skin cancer and other skin lesions. The mechanism of the technique is first to improve the images and then segment the lesion area. CNN models are fed lesion area images of the ISIC 2019 dataset for feature map extraction through convolutional layers and pooling. To keep important features and delete redundant features by PCA, the feature vectors generated by PCA are sent to ANNs and RF networks for training and performance testing.

Hybrid models based on fused features of CNN models for image analysis of skin lesions of the ISIC 2019 dataset have high capabilities in distinguishing skin cancer from other skin diseases.

**Figure 13:**
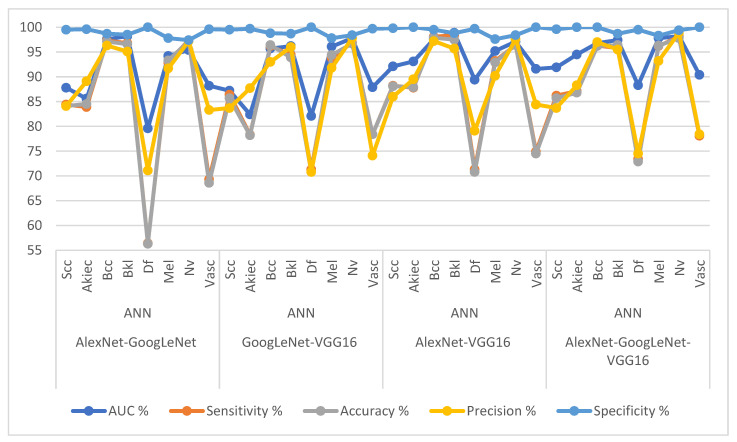
Display Performance results of CNN-ANN hybrid models based on the fused CNN models for early diagnosis and discrimination of skin cancer and other skin lesions.

Table 7 and Figure 13 present the measurement performance of the CNN-ANN hybrid models based on the fused CNN models for image analysis of the ISIC 2019 dataset for early diagnosis of skin lesions as follows: the AlexNet-GoogLeNet-ANN model achieved average results: an AUC of 90.83%, sensitivity of 84.8%, accuracy of 95%, precision of 88.49%, and specificity of 98.89%. The GoogLeNet-VGG16-ANN model achieved average results: an AUC of 90.68%, sensitivity of 86.96%, accuracy of 94.6%, precision of 86.86%, and specificity of 87.83%. The AlexNet-VGG16-ANN model achieved average results: an AUC of 94.38%, sensitivity of 88.54%, accuracy of 95.2%, precision of 89.96%, and specificity of 99.23%. The AlexNet-GoogLeNet-VGG16-ANN model yielded average results: an AUC of 94.41%, sensitivity of 88.90%, accuracy of 96.10%, precision of 88.69%, and specificity of 99.44%.

**Figure 14:**
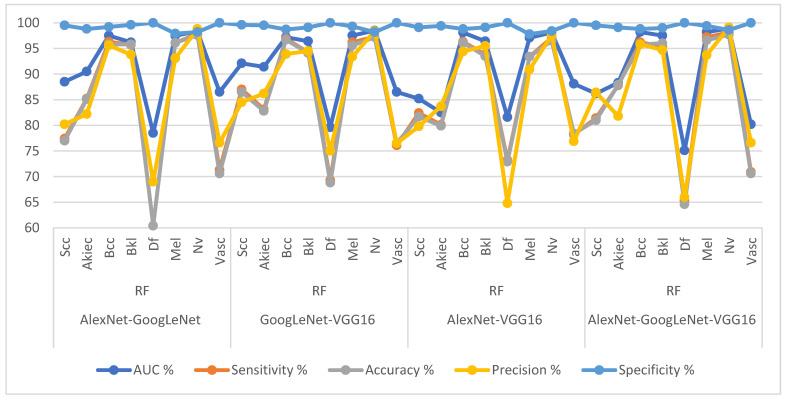
Display Performance results of CNN-RF hybrid models based on the fused CNN models for early diagnosis and discrimination of skin cancer and other skin lesions.

Table 8 and Figure 14 present the measurement performance of the CNN-ANN hybrid models based on the fused CNN models for image analysis of the ISIC 2019 dataset for the early diagnosis of skin lesions as follows: the AlexNet-GoogLeNet-RF model achieved average results: an AUC of 91.69%, sensitivity of 85.03%, accuracy of 95.3%, precision of 86.16%, and specificity of 99.15%. The GoogLeNet-VGG16-RF model achieved average results: an AUC of 92.40%, sensitivity of 87.51%, accuracy of 95.30%, precision of 87.80%, and specificity of 99.30%. The AlexNet-VGG16-RF model achieved average results: an AUC of 90.89%, sensitivity of 86.81%, accuracy of 94.30%, precision of 85.45%, and specificity of 99.08%. The AlexNet-GoogLeNet-VGG16-RF model yielded average results: an AUC of 90.33%, sensitivity of 86.54%, accuracy of 95.70%, precision of 86.76%, and specificity of 99.30%.

The hybrid models of CNN-ANN and RF-CNN based on fusion features of the CNN model produce confusion matrices that show the performance of the hybrid models for the early detection of skin cancer for distinguishing skin cancer from other lesions.

**Figure 15:**
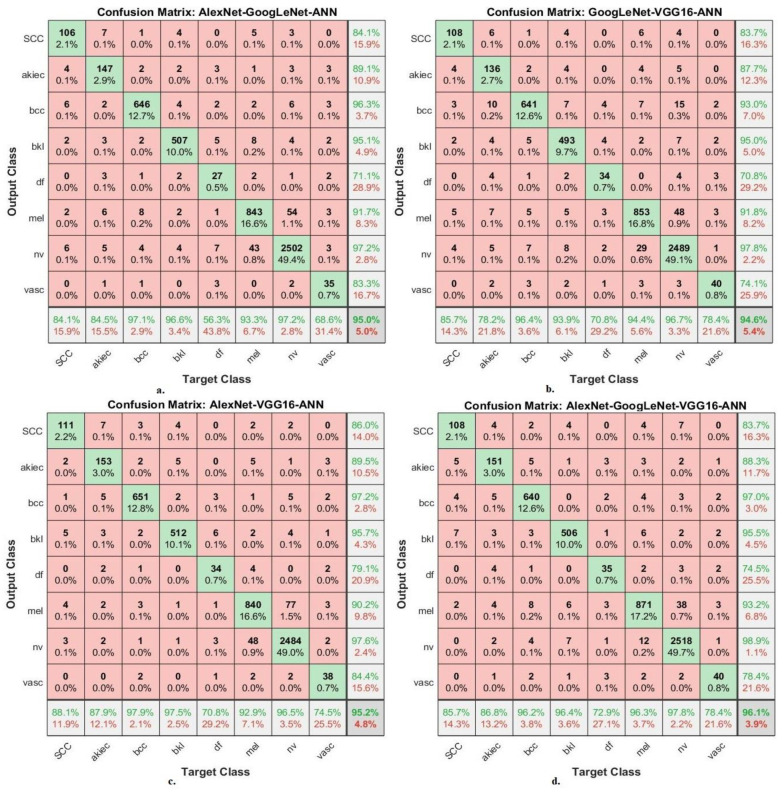
Confusion matrix for displaying performance results of CNN-ANN hybrid models based on the fused CNN models for early diagnosis and discrimination of skin cancer and other skin lesions (**a**) AlexNet-GoogLeNet-ANN (**b**) GoogLeNet-VGG16-ANN (**c**) AlexNet-VGG16-ANN (**d**) AlexNet-GoogLeNet-VGG16-ANN.

Figure 15 presents the confusion matrix for the AlexNet-GoogLeNet-ANN, GoogLeNet-VGG16-ANN, AlexNet-VGG16-ANN, and AlexNet-GoogLeNet-VGG16-ANN models for the early diagnosis of skin lesions of the ISIC 2019 dataset. The figure shows the accuracy of each class, where the AlexNet-GoogLeNet-ANN model reached an accuracy for each class as follows: for Scc class of 84.1%, for Akice class of 84.5%, for Bcc class of 97.1%, for Bkl class of 96.6%, for Df class of 56.3%, for Mel class of 93.3%, for Nv class of 97.2%, and Vasc class of 68.6%. The GoogLeNet-VGG16-ANN model achieved the accuracy for each class as follows: for Scc class of 85.7%, for Akice class of 78.2%, for Bcc class of 96.4%, for Bkl class of 93.9%, for Df class of 70.8%, for Mel class of 94.4%, for Nv class of 96.7%, and Vasc class of 78.4%. The AlexNet-VGG16-ANN model resulted in accuracy for each class as follows: for Scc class of 88.1%, for Akice class of 87.9%, for Bcc class of 97.9%, for Bkl class of 97.5%, for Df class of 70.8%, for Mel class of 92.9%, for Nv class of 96.5%, and Vasc class of 74.5%. The AlexNet-GoogLeNet-VGG16-ANN model resulted in accuracy for each class as follows: for Scc class of 85.7%, for Akice class of 86.8%, for Bcc class of 96.2%, for Bkl class of 96.4%, for Df class of 72.9%, for Mel class of 96.3%, for Nv class of 97.8%, and Vasc class of 78.4%.

**Figure 16:**
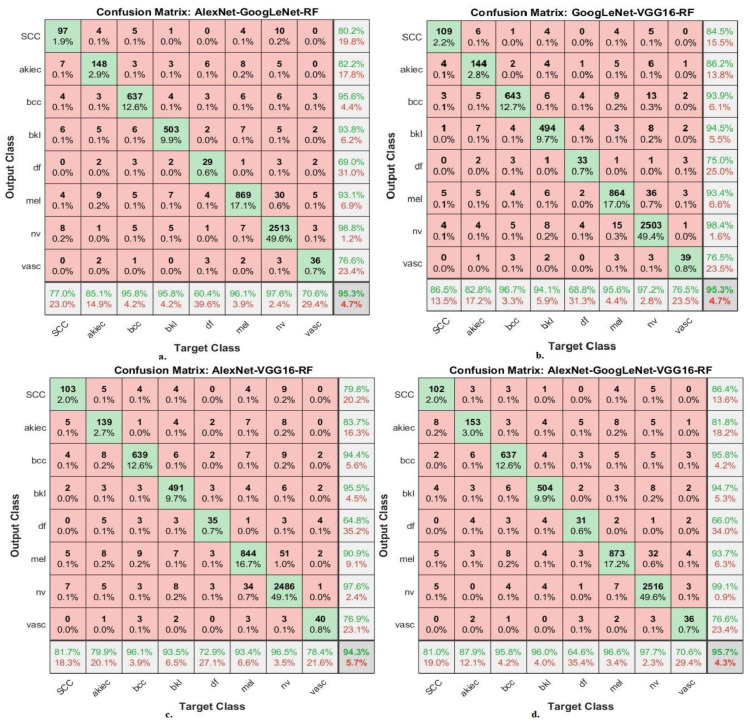
Confusion matrix for displaying performance results of CNN-RF hybrid models based on the fused CNN models for early diagnosis and discrimination of skin cancer and other skin lesions (**a**) AlexNet-GoogLeNet-RF (**b**) GoogLeNet-VGG16-RF (**c**) AlexNet-VGG16-RF (**d**) AlexNet-GoogLeNet-VGG16-RF.

Figure 16 presents the confusion matrix for the AlexNet-GoogLeNet-RF, GoogLeNet-VGG16-RF, AlexNet-VGG16-RF, and AlexNet-GoogLeNet-VGG16-RF models for the early diagnosis of skin lesions of the ISIC 2019 dataset. The figure shows the accuracy of each class, where the AlexNet-GoogLeNet-RF model reached to an accuracy for each class as follows: for Scc class of 77%, for Akice class of 85.1%, for Bcc class of 95.8%, for Bkl class of 95.8%, for Df class of 60.4%, for Mel class of 96.1%, for Nv class of 97.6%, and Vasc class of 70.6%. The GoogLeNet-VGG16-RF model achieved the accuracy for each class as follows: for Scc class of 86.5%, for Akice class of 82.8%, for Bcc class of 96.7%, for Bkl class of 94.1%, for Df class of 68.8%, for Mel class of 95.6%, for Nv class of 97.2%, and Vasc class of 76.5%. The AlexNet-VGG16-RF model resulted in accuracy for each class as follows: for Scc class of 81.7%, for Akice class of 79.9%, for Bcc class of 96.1%, for Bkl class of 93.5%, for Df class of 72.9%, for Mel class of 93.4%, for Nv class of 96.5%, and Vasc class of 78.4%. The AlexNet-GoogLeNet-VGG16-RF model resulted in accuracy for each class as follows: for Scc class of 81%, for Akice class of 87.9%, for Bcc class of 95.8%, for Bkl class of 96%, for Df class of 64.6%, for Mel class of 96.6%, for Nv class of 97.7%, and Vasc class of 70.6%.

## 5. Discussion and Comparison of the Performance Results of the Systems

Exposure of the skin to ultraviolet radiation causes DNA damage to skin cells and skin cancer. Melanoma is one of the deadliest skin lesions, leading to death if not diagnosed and treated early. Many skin lesions have similar clinical characteristics and vital signs, especially in the initial stages, which require highly experienced dermatologists. Automated systems help diagnose and distinguish skin cancer from other skin lesions. This study focuses on developing several hybrid systems based on fused CNN features. ISIC 2019 dataset images were optimized, and the GAC method segmented and isolated the lesion area from healthy skin.

The first methodology for the early recognition of skin cancer from other skin lesions involved using the pre-trained AlexNet, GoogLeNet and VGG16 models. The results reached by the pre-trained models were not satisfactory, especially for classifying some types of pests. The pre-trained AlexNet, GoogLeNet, and VGG16 models achieved an accuracy of 88.5%, 88.7%, and 88.3%, respectively.

The second methodology for diagnosing ISIC 2019 images and distinguishing skin cancer from other skin lesions involved using AlexNet, GoogLeNet and VGG16 models based on the GAC algorithm, in which the lesion area was segmented from the images and fed to AlexNet, GoogLeNet and VGG16 models. When feeding the CNN models with lesion areas only, the results are improved, as the AlexNet, GoogLeNet, and VGG16 models reached an accuracy of 92.2%, 91.8%, and 90.5%, respectively.

The third methodology for the early diagnosis of skin cancer and distinguishing them from other lesions involved using the hybrid models CNN-ANN and CNN-RF based on the GAC algorithm. The hybrid models of AlexNet-ANN, GoogLeNet-ANN, and VGG16-ANN achieved an accuracy of 94.8%, 93.7%, and 93.6%, respectively, while the hybrid models of AlexNet-RF, GoogLeNet-RF, and VGG16-RF achieved an accuracy of 94.3%, 94.9%, and 94.2%, respectively.

The fourth methodology for the early diagnosis of skin cancer and distinguishing them from other lesions involved using hybrid models between fused CNN models and ANN and RF networks. CNN features were combined and classified by ANN and RF networks. The hybrid models AlexNet-GoogLeNet-ANN, GoogLeNet-VGG16-ANN, AlexNet-VGG16-ANN and AlexNet-GoogLeNet-VGG16-ANN achieved accuracy of 95%, 94.6%, 95.2% and 96.1%, while the hybrid models AlexNet-GoogLeNet-RF, GoogLeNet-VGG16-RF, AlexNet-VGG16-RF and AlexNet-GoogLeNet-VGG16-RF achieved accuracy of 95.3%, 95.3%, 94.3% and 95.7%.

**Figure 17:**
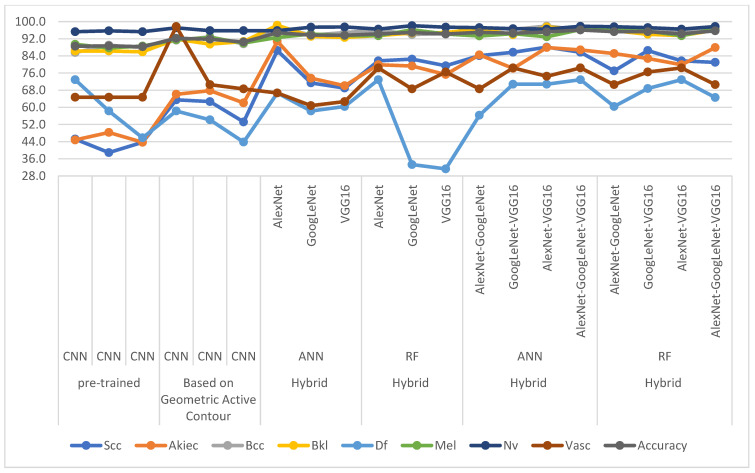
Performance of the proposed systems for dermatoscopic image analysis for early diagnosis of skin cancer for the ISIC 2019 data set and distinguishing them from other skin lesions.

Table 9 and Figure 17 present the performance of all systems for analyzing dermatoscopic images for diagnosing skin cancer from the ISIC 2019 data set and distinguishing them from other skin lesions. The table discusses the overall accuracy and accuracy of each type of skin lesion in the ISIC 2019 dataset. The classification of skin lesions was carried out by three pre-trained models, AlexNet, GoogLeNet and VGG16, which did not achieve good results, especially for classifying some classes (lesions). When applying the GAC algorithm to segment the lesion area and feeding it to the AlexNet, GoogLeNet and VGG16 models, the results improved better than when providing the models with the whole picture. CNN-ANN and CNN-RF hybrid models based on the GAC algorithm have been implemented. It is noted that the classification results of the ISIC 2019 data set by the hybrid technique are better than the pre-trained models. Due to the similar characteristics of skin lesions and to achieve promising accuracy, CNN-ANN and CNN-RF hybrid models were applied based on fused CNN features. It is noted that the ANN and RF networks with combined CNN features, after deleting the non-significant and repetitive features by PCA, achieved the best results compared to other strategies.

The improvement in the accuracy of each class is noted as follows: for the Scc class, the accuracy improved from 38.9% by the pre-trained GoogLeNet to 88.1% by the AlexNet-VGG16-ANN hybrid model. The accuracy of the Akiec class improved from 43.7% by the pre-trained VGG16 to 90.8% by the AlexNet-ANN hybrid model. The accuracy of the Bcc class improved from 85.6% by the pre-trained VGG16 to 97.9% by the AlexNet-VGG16-ANN hybrid model. The accuracy of the Bkl class improved from 85.9% by the pre-trained VGG16 to 98.3% by the AlexNet-ANN hybrid model. Class Df accuracy improved from 43.8% by the pre-trained VGG16 to 97.9% by the AlexNet-VGG16-RF hybrid model. The accuracy of the Mel class improved from 87.7% by the pre-trained VGG16 to 97.9% by the AlexNet-GoogLeNet-VGG16-RF hybrid model. Class Nv accuracy improved from 95.3% by the pre-trained VGG16 to 98.1% by the GoogLeNet-RF hybrid model. The accuracy of the Vasc class improved from 64.7% by the pre-trained CNN to 78.4% by the AlexNet-GoogLeNet-VGG16-ANN hybrid model.

## 6. Conclusions

Many skin lesions are similar in the early stages, making it difficult to distinguish skin cancer from other skin lesions. Thus, many hybrid systems with fused features have been developed based on segmentation of lesion areas and their isolation from the rest of the healthy skin. The images were optimized, and the lesion area was segmented by the GAC algorithm and fed into AlexNet, GoogLeNet and VGG16 models. The first hybrid model involved dermatoscopic image analysis for the early diagnosis of skin cancer using the ISIC 2019 data set and their distinction from other skin lesions using CNN-ANN and CNN-RF. The second hybrid model for diagnosing ISIC 2019 dataset images involved the hybrid model based on fused CNN features. The AlexNet-GoogLeNet-VGG16-ANN hybrid model is based on extracting the features from the AlexNet-GoogLeNet-VGG16 models separately and reducing the dimensions by eliminating the redundant and unimportant features by PCA, then fusing the features of the three models serially and sending them to a network ANN for classification. The AlexNet-GoogLeNet-VGG16-ANN hybrid model achieved an AUC of 94.41%, sensitivity of 88.90%, accuracy of 96.10%, precision of 88.69%, and specificity of 99.44%.

This study aims to develop high-efficiency systems to help physicians diagnose skin diseases and differentiate between skin cancer and other lesions.

The limitation in this method is the imbalance of the data set, which is processed by the data augmentation technique.

Future work will involve developing systems to classify the ISIC 2019 dataset using fusion features between handcrafted features and CNN models and generalizing the proposed systems to the ISIC 2020 dataset.

## Figures and Tables

**Table 1:** Splitting the ISIC 2019 data sets of skin cancer.

Phase	80% (80:20)		Testing 20%
Classes	Training (80%)	Validation (20%)	
Squamous cell carcinoma (Scc)	402	100	126
Actinic keratoses (Akiec)	555	139	173
Basal cell carcinoma (Bcc)	2126	532	665
Benign keratosis lesions (Bkl)	1679	420	525
Dermatofibroma (Df)	153	38	48
Melanoma (Mel)	2894	724	904
Melanocytic nevi (Nv)	8240	2060	2575
Vascular (Vasc)	162	40	51

**Table 2:** Balancing dermoscopic image of ISIC 2019 dataset of skin lesions.

Phase	Training Dataset							
Classes	Scc	Akiec	Bcc	Bkl	Df	Mel	Nv	Vasc
Bef-augm	402	555	2126	1679	153	2894	8240	162
Aft-augm	8040	8325	8504	8395	6120	8682	8240	6480

**Table 3:** Performance results of pre-trained CNN models for analysis of the ISIC 2019 dataset image for early diagnosis and differentiation of skin cancer and other skin lesions.

Models	Type of Lesion	AUC %	Sensitivity %	Accuracy %	Precision %	Specificity %
AlexNet	Scc	50.2	45.4	45.2	54.8	98.5
	Akiec	51.5	46.3	44.8	57.4	99.2
	Bcc	89.3	86.3	85.6	84.3	97.6
	Bkl	88.1	85.9	86.3	84.7	97.9
	Df	84.9	72.8	72.9	68.6	99.6
	Mel	91.2	89.2	89.5	85.9	97.4
	Nv	94.8	95.2	95.3	95.2	94.8
	Vasc	78.6	65.2	64.7	68.8	99.6
GoogLeNet	Scc	46.5	39.3	38.9	49	99.1
	Akiec	88.2	87.9	48.3	57.9	98.4
	Bcc	90.4	89.2	89	84	96.9
	Bkl	87.6	86.4	86.3	82.8	97.5
	Df	68.2	58.2	58.3	66.7	99.5
	Mel	89.3	87.7	87.7	89	98.1
	Nv	97.1	95.8	95.7	94.9	95.4
	Vasc	72.5	65.3	64.7	78.7	99.6
VGG16	Scc	51.5	44.2	43.7	55	99.2
	Akiec	52.3	44.3	43.7	56.7	98.7
	Bcc	89.6	87.9	87.8	83.2	97.3
	Bkl	88.3	86.2	85.9	85.6	97.9
	Df	59.8	46.4	45.8	50	99.6
	Mel	92.9	88.7	88.6	86.4	96.7
	Nv	96.6	94.6	95.3	94.6	93.8
	Vasc	77.2	65.4	64.7	80.5	98.6

**Table 4:** Performance results of pre-trained CNN models based on ROI using GAC method for early diagnosis and differentiation of skin cancer and other skin lesions.

Models	Type of Lesion	AUC %	Sensitivity %	Accuracy %	Precision %	Specificity %
AlexNet	Scc	70.2	63.4	63.5	69	99.1
	Akiec	72.4	66.2	66.1	71	98.6
	Bcc	92.3	90.8	91.3	92.5	98.7
	Bkl	93.8	91.7	91.8	90.3	99.4
	Df	68.9	58.3	58.3	54.9	99.9
	Mel	92.5	91.2	91.5	92	97.7
	Nv	98.1	96.9	97.1	96.1	96.3
	Vasc	75.2	65.2	64.7	67.3	99.5
GoogLeNet	Scc	69.1	63.1	62.7	67.5	99.2
	Akiec	75.6	68.3	67	76.6	98.8
	Bcc	94.6	92.8	92.9	89.8	97.6
	Bkl	93.4	90.4	89.5	90.4	99.3
	Df	66.2	54.3	54.2	56.5	99.8
	Mel	94.2	92.7	92.6	90.2	97.5
	Nv	97.1	95.8	95.8	96.3	96.4
	Vasc	84.6	70.9	70.6	69.2	99.7
VGG16	Scc	62.2	53.4	53.2	54.9	99.3
	Akiec	67.1	62.3	62.1	69.7	98.5
	Bcc	92.3	89.8	89.6	88.8	97.8
	Bkl	94.2	91.4	90.7	88	98.6
	Df	50.2	44.2	43.8	60	99.5
	Mel	92.9	89.7	90	89.5	97.7
	Nv	97.5	95.6	95.8	95.7	96.2
	Vasc	98.6	70.2	68.6	62.5	99.7

**Table 5:** Performance results of CNN-ANN hybrid models based on ROI using the GAC method for early diagnosis and differentiation of skin cancer and other skin lesions.

Hybrid-Models	Type of Lesion	AUC %	Sensitivity %	Accuracy %	Precision %	Specificity %
AlexNet-ANN	Scc	89.1	87.2	86.5	88.6	99.6
	Akiec	92.3	91.2	90.8	88.8	99.5
	Bcc	95.2	98.4	97.6	96.9	100
	Bkl	96.7	97.8	98.3	96.1	99.6
	Df	75.1	66.9	66.7	72.7	99.5
	Mel	95.2	93.4	92.6	88.5	96.8
	Nv	97.1	96.1	95.8	97.5	98.1
	Vasc	74.2	67.4	66.7	82.9	100
GoogLeNet-ANN	Scc	82.5	71.2	71.4	67.7	99.2
	Akiec	88.8	73.8	73.6	75.7	99.4
	Bcc	96.7	93.9	93.7	94.4	98.8
	Bkl	96.4	93.2	93.1	93.3	98.5
	Df	67.2	58.4	58.3	57.1	100
	Mel	96.1	93.7	94.2	92.3	97.8
	Nv	98.4	97.1	97.4	97.7	98.2
	Vasc	92.8	91.4	60.8	70.5	100
VGG16-ANN	Scc	77.2	69.4	69	70.2	98.8
	Akiec	81.2	70.2	70.1	74.8	98.6
	Bcc	95.6	94.7	94.9	93.2	99.3
	Bkl	93.1	92.9	92.6	92.7	99.1
	Df	64.5	60.2	60.4	63	100
	Mel	94.2	93.4	93.5	93.6	99.1
	Nv	98.1	97.7	97.5	97.4	96.8
	Vasc	66.6	62.9	62.7	61.5	99.8

**Table 6:** Performance results of CNN-RF hybrid models based on ROI using the GAC method for early diagnosis and differentiation of skin cancer and other skin lesions.

Hybrid-Models	Type of Lesion	AUC %	Sensitivity %	Accuracy %	Precision %	Specificity %
AlexNet-RF	Scc	89.2	82.3	81.7	79.8	99.1
	Akiec	84.6	80.4	79.9	83.7	99.3
	Bcc	96.7	95.7	96.1	94.4	89.7
	Bkl	95.3	93.8	93.5	95.5	98.5
	Df	87.1	73.4	72.9	64.8	99.6
	Mel	93.1	92.9	93.4	90.9	97.8
	Nv	94.5	96.6	96.5	97.6	98.4
	Vasc	81.9	78.4	78.4	76.9	99.7
GoogLeNet-RF	Scc	90.2	83.4	82.5	75.4	98.7
	Akiec	82.1	79.3	79.3	81.2	99.2
	Bcc	94.3	94.3	94	95.6	98.6
	Bkl	91.2	94.8	94.7	94.3	99.1
	Df	79.6	33.4	33.3	59.3	99.8
	Mel	94.2	96.4	96.2	92.1	97.5
	Nv	96.8	97.8	98.1	98.7	99.5
	Vasc	85.4	69.3	68.6	74.5	100
VGG16-RF	Scc	82.5	79.3	79.4	76.9	99.2
	Akiec	86.2	75.2	75.3	79.9	98.8
	Bcc	96.1	94.3	94.4	93.3	98.6
	Bkl	94.5	95.4	94.9	94.9	98.5
	Df	67.2	31.4	31.3	50	100
	Mel	92.9	94.3	94.2	88.8	97.2
	Nv	97.2	96.9	97.4	98.9	99.3
	Vasc	87.2	76.4	76.5	79.6	99.7

**Table 7:** Performance results of CNN-ANN hybrid models based on the fused CNN models for early diagnosis and differentiation of skin cancer and other skin lesions.

Fusion Features	Classifier	Type of Lesion	AUC %	Sensitivity %	Accuracy %	Precision %	Specificity %
AlexNet-GoogLeNet	ANN	Scc	87.8	84.4	84.1	84.1	99.5
		Akiec	85.6	83.9	84.5	89.1	99.6
		Bcc	97.6	97.3	97.1	96.3	98.7
		Bkl	98.2	96.8	96.6	95.1	98.5
		Df	79.6	56.4	56.3	71.1	100
		Mel	94.2	92.9	93.3	91.7	97.8
		Nv	95.4	97.4	97.2	97.2	97.4
		Vasc	88.2	69.3	68.6	83.3	99.6
GoogLeNet-VGG16	ANN	Scc	87.2	86.4	85.7	83.7	99.5
		Akiec	82.4	78.3	78.2	87.7	99.7
		Bcc	95.7	96.2	96.4	93	98.8
		Bkl	96.2	94.4	93.9	96	98.7
		Df	82.1	71.3	70.8	70.8	100
		Mel	96.1	93.8	94.4	91.8	97.8
		Nv	97.8	96.9	96.7	97.8	98.4
		Vasc	87.9	78.4	78.4	74.1	99.7
AlexNet-VGG16	ANN	Scc	92.1	88.2	88.1	86	99.8
		Akiec	93.1	87.8	87.9	89.5	100
		Bcc	97.6	98.1	97.9	97.2	99.5
		Bkl	98.9	98.4	97.5	95.7	98.8
		Df	89.4	71.3	70.8	79.1	99.7
		Mel	95.2	93.2	92.9	90.2	97.6
		Nv	97.1	96.4	96.5	97.6	98.4
		Vasc	91.6	74.9	74.5	84.4	100
AlexNet-GoogLeNet-VGG16	ANN	Scc	91.9	86.2	85.7	83.7	99.6
		Akiec	94.5	87.1	86.8	88.3	100
		Bcc	96.8	96.3	96.2	97	100
		Bkl	97.4	95.8	96.4	95.5	98.7
		Df	88.3	73.4	72.9	74.5	99.5
		Mel	97.8	96.2	96.3	93.2	98.3
		Nv	98.2	98.1	97.8	98.9	99.4
		Vasc	90.4	78.1	78.4	78.4	100

**Table 8:** Performance results of CNN-RF hybrid models based on the fused CNN models for early diagnosis and differentiation of skin cancer and other skin lesions.

Fusion Features	Classifier	Type of Lesion	AUC %	Sensitivity %	Accuracy %	Precision %	Specificity %
AlexNet-GoogLeNet	RF	Scc	88.5	77.4	77	80.2	99.5
		Akiec	90.5	85.2	85.1	82.2	98.8
		Bcc	97.5	96.3	95.8	95.6	99.2
		Bkl	96.2	95.7	95.8	93.8	99.6
		Df	78.5	60.4	60.4	69	100
		Mel	97.5	96.1	96.1	93.1	97.9
		Nv	98.3	97.8	97.6	98.8	98.2
		Vasc	86.5	71.3	70.6	76.6	100
GoogLeNet-VGG16	RF	Scc	92.1	87	86.5	84.5	99.6
		Akiec	91.4	83.1	82.8	86.2	99.5
		Bcc	97.2	96.9	96.7	93.9	98.7
		Bkl	96.4	94.2	94.1	94.5	99.1
		Df	79.6	69.3	68.8	75	100
		Mel	97.5	96.2	95.6	93.4	99.3
		Nv	98.5	97.3	97.2	98.4	98.2
		Vasc	86.5	76.1	76.5	76.5	100
AlexNet-VGG16	RF	Scc	85.2	82.4	81.7	79.8	99.1
		Akiec	82.5	80.1	79.9	83.7	99.4
		Bcc	98.1	96.3	96.1	94.4	98.8
		Bkl	96.4	93.8	93.5	95.5	99.1
		Df	81.6	73.2	72.9	64.8	100
		Mel	97.1	93.4	93.4	90.9	97.8
		Nv	98.1	97.1	96.5	97.6	98.4
		Vasc	88.1	78.2	78.4	76.9	100
AlexNet-GoogLeNet-VGG16	RF	Scc	86.2	81.4	81	86.4	99.5
		Akiec	88.3	87.8	87.9	81.8	99.1
		Bcc	98.2	96.2	95.8	95.8	98.8
		Bkl	97.5	95.4	96	94.7	99
		Df	75.1	65.3	64.6	66	100
		Mel	98.2	97.2	96.6	93.7	99.4
		Nv	98.9	98.1	97.7	99.1	98.6
		Vasc	80.2	70.9	70.6	76.6	100

**Table 9:** Performance of proposed systems for dermatoscopic image analysis for early diagnosis of skin cancer for the ISIC 2019 dataset and distinguishing them from other skin lesions.

Techniques		Features	Scc	Akiec	Bcc	Bkl	Df	Mel	Nv	Vasc	Accuracy
Pre-trained	AlexNet		45.2	44.8	85.6	86.3	72.9	89.4	95.3	64.7	88.5
	GoogLeNet		38.9	48.3	89.0	86.3	58.3	87.7	95.7	64.7	88.7
	VGG16		43.7	43.7	87.8	85.9	45.8	88.6	95.3	64.7	88.3
Based on Geometric Active Contour	AlexNet		63.5	66.1	91.3	91.8	58.3	91.5	97.1	97.7	92.2
	GoogLeNet		62.7	67.8	92.9	89.5	54.2	92.6	95.8	70.6	91.8
	VGG16		53.2	62.1	89.6	90.7	43.8	90.0	95.8	68.6	90.5
Hybrid	ANN	AlexNet	86.5	90.8	97.6	98.3	66.7	92.6	95.8	66.7	94.8
		GoogLeNet	71.4	73.6	93.7	93.1	58.3	94.2	97.4	60.8	93.7
		VGG16	69.0	70.1	94.9	92.6	60.4	93.5	97.5	62.7	93.6
Hybrid	RF	AlexNet	81.7	79.9	96.1	93.5	72.9	93.4	96.5	78.4	94.3
		GoogLeNet	82.5	79.3	94.0	94.7	33.3	96.2	98.1	68.6	94.9
		VGG16	79.4	75.3	94.4	94.9	31.3	94.2	97.4	76.5	94.2
Hybrid	ANN	AlexNet-GoogLeNet	84.1	84.5	97.1	96.6	56.3	93.3	97.2	68.6	95.0
		GoogLeNet-VGG16	85.7	78.2	96.4	93.9	70.8	94.4	96.7	78.4	94.6
		AlexNet-VGG16	88.1	87.9	97.9	97.5	70.8	92.9	96.5	74.5	95.2
		AlexNet-GoogLeNet-VGG16	85.7	86.8	96.2	96.4	72.9	96.3	97.8	78.4	96.1
Hybrid	RF	AlexNet-GoogLeNet	77.0	85.1	95.8	95.8	60.4	96.1	97.6	70.6	95.3
		GoogLeNet-VGG16	86.5	82.8	96.7	94.1	68.8	95.6	97.2	76.5	95.3
		AlexNet-VGG16	81.7	79.9	96.1	93.5	72.9	93.4	96.5	78.4	94.3
		AlexNet-GoogLeNet-VGG16	81.0	87.9	95.8	96.0	64.6	96.6	97.7	70.6	95.7

## Data Availability

In this work, the data supporting the performance of the proposed systems were obtained from the ISIC 2019 dataset that is publicly available at the following link: https://challenge.isic-archive.com/data/#2019 (accessed on 18 October 2022).
